# Establishment of *Babesia bovis* In Vitro Culture Using Medium Free of Animal Products

**DOI:** 10.3390/pathogens10060770

**Published:** 2021-06-19

**Authors:** Jesús A. Álvarez Martínez, Julio V. Figueroa Millán, Massaro W. Ueti, Carmen Rojas-Martínez

**Affiliations:** 1 Babesia Unit-CENID-Salud Animal e Inocuidad, INIFAP, Carr. Fed. Cuernavaca-Cuautla No. 8534, Col. Progreso, Jiutepec, Morelos C.P. 62550, Mexico; alvarez.jesus@inifap.gob.mx (J.A.Á.M.); figueroa.julio@inifap.gob.mx (J.V.F.M.); 2 Agricultural Research Service-Animal Disease Research Unit, The US Department of Agriculture, Pullman, WA 99164, USA

**Keywords:** *Babesia bovis*, in vitro culture, animal component-free medium, mixture lipids, perfusion bioreactor

## Abstract

*Babesia bovis*, an etiological agent of bovine babesiosis, causes a significant burden to the cattle industry worldwide. The most efficient method to mitigate bovine babesiosis is a live vaccine produced by serial passage in splenectomized cattle. However, there are several concerns regarding live vaccine production, including variation between batches and the use of many animals. In this study, we report a *B. bovis*-SF strain continuously cultured in a medium free of components of animal origin enriched with a chemically defined lipid mixture (CD lipid mixture) and the use of a perfusion bioreactor to harvest a large amount of *B. bovis*. Six culture media were compared, including VP-SFM, CD-CHO, CD-Hydrolyzed, CD-CHO, SFM, and ADMEM/F12. We found that the VP-SFM medium performed the best for *B. bovis* growth, with a maximum percentage of parasitized erythrocytes (PPE) of 8.6%. The effect of six dilutions of a commercial mixture of CD lipids added to VP-SFM showed that the CD lipid mixture at a dilution of 1:100 had the best *B. bovis* growth curve, with a maximum PPE of 13.9%. Propagation of the in vitro *B. bovis* culture was scaled up in a perfusion bioreactor using VP-SFM with a CD lipid mixture, and the PPE reached over 32%. The continuous in vitro *B. bovis* culture in a medium free of animal origin components could potentially reduce and replace the use of animals to produce a reagent for diagnostics and live vaccines to control bovine babesiosis.

## 1. Introduction

The phylum Apicomplexa comprises intracellular protozoan parasites of enormous global importance in the medical and veterinary fields [1]. *Babesia bovis* and *B. bigemina* are obligate intraerythrocytic parasites that cause bovine babesiosis in tropical and subtropical regions [2,3], and both parasites are transmitted by *Rhipicephalus microplus* or *R. annulatus*. Bovine babesiosis causes great economic losses for the livestock industry [4,5]. *Babesia bovis* is more virulent and causes higher mortality than *B. bigemina* [6,7]. Acute *B. bovis* infection is typically characterized by fever (40–41 °C), neurological signs, anemia, respiratory distress syndrome, multi-organ failure, and often death [8].

Understanding *B. bovis* cycling in the erythrocytes is critically important to mitigate bovine babesiosis. Continuous *B. bovis* cultivation in a microaerophilic stationary phase (MASP) was developed and facilitated, studying *B. bovis* in vitro growth, including nutritional needs for growth of *Babesia* spp. [9,10,11], drug evaluation [12,13,14,15], identification of proteins involved in attachment and the invasion process [16], antigen discovery [17,18], recovering or increasing the percentage of parasitized erythrocytes (PPE) of *Babesia* from infected animals [19], and genetic modification of *Babesia* parasites [20,21].

Traditionally, in vitro *Babesia* culture consists of a suspension of adult bovine erythrocytes in M-199 medium with Earle’s salt supplemented with 40% bovine serum [22,23]. The medium contains essential components for *B. bovis* proliferation, including nutrients, growth factors, and hormones, and regulates pH and osmotic pressure [10]. Previous modifications to the culture medium included reducing or removing bovine serum and replacing it with Albumax^®^ I and II for in vitro growth of *B. divergens* and *B. bovis* [24,25]. Additionally, hypoxanthine was added to the culture medium for *B. bovis* and *B. bigemina* growth, which allowed a reduction in the bovine serum concentration [26]. Subsequently, important innovations were made to eliminate bovine serum for continuous cultivation of *B. bovis*. Advanced DMEM/F12 (ADMEM/F12) medium supplemented with insulin-transferrin-selenite and putrescine was used [10,11]. Unfortunately, ADMEM/F12 medium contains components of animal origin. The Food and Drug Administration (FDA) has recommended that in the production of viral vaccines, bovine serum and undefined supplements of animal origin are eliminated from the culture medium to remove the risk of contamination by adventitious agents and to obtain a greater reliability of the vaccines [14]. Different chemically defined culture media have been developed with components free of animal origin. The media were designed to grow several cell lines of interest, particularly to produce viral vaccines [27,28,29,30]. Likewise, a culture medium without animal components, VP-SFM AGT™ (VP-SFM), allowed the successful growth of *Toxoplasma gondii*, as well as *B. bigemina*, in vitro [31,32]. In other studies, it has been indicated that fatty acids and lipids are critical in culture media without bovine serum supplements to provide energy, and constituents of cell membranes involved in transport and signaling pathways [33]. Specifically, for *Babesia* spp., lipids are essential components of the culture medium [24]. For instance, *B. divergens* needs phospholipids for growth that the parasites obtain from an external source. *Babesia caballi* proliferated in a serum-free medium supplemented with lipids, hypoxanthine, and Albumax I [34]. Recently, Alvarez et al. (2020) demonstrated continuous in vitro proliferation of *B. bigemina* in culture medium VP-SFM supplemented with a CD lipid mixture at the small and large scales [32]. Nevertheless, VP-SFM has never been evaluated for the in vitro culture of *B. bovis*. The development of new methodologies to reduce the cost of biological material required for diagnostics and vaccines for bovine babesiosis is highly desirable. In the present study, we evaluated different culture media free of animal components. We determined the optimal concentration of a CD lipid mixture for large-scale in vitro proliferation of *B. bovis*.

## 2. Results

### 2.1. Optimal Culture Medium Using Animal Component-Free Medium to Grow B. bovis-SF In Vitro

**Figure 1:**
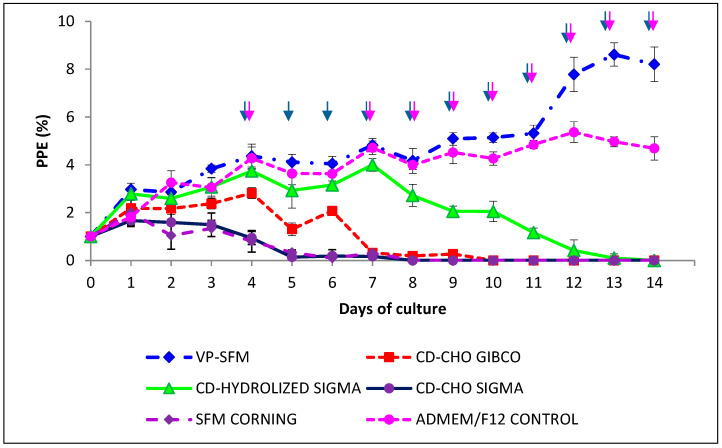
Medium VP-SFM AGT™ (VP-SFM) without animal products supports in vitro proliferation of *B. bovis* (*B. bovis*-SF). Five media without animal products were compared to an ADME/F12 containing animal products. Values represent the mean from three separate experiments using triplicate wells for each test condition. PPE: percentage of parasitized erythrocytes. Arrows indicate subculturing performed when PPE was above 4% and adjusted back to 1%.

Among the six media without animal origin components for growing *B. bovis* in vitro, VP-SFM performed the best. Culture with ADMEM/F12 showed similar parasite growth to VP-SFM until day 11. Although there was no statistical difference between these two means (*p* > 0.05), a biologically higher PPE was observed with VP-SFM than with ADMEM/F12 (8.6% vs. 5.3%). The other media failed to support the growth of *B. bovis* (Figure 1).

**Figure 2:**
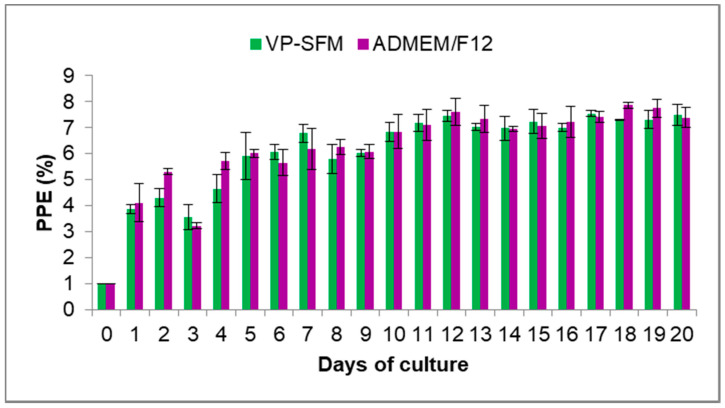
VP-SFM supports long-term continuous in vitro culture of *B bovis*. Values represent the mean from three separate experiments using triplicate wells for each test condition. PPE: percentage of parasitized erythrocytes. Subculturing was performed when PPE was above 4% and adjusted back to 1%.

The VP-SFM medium was evaluated for 20 days in culture to determine if the medium supported the continuous growth of *B. bovis* as compared to the ADMEM/F12 medium. The maximum PPE values were 7.5% and 7.8% for VP-SFM and ADMEM/F12, respectively. There was no statistically significant difference (*p* > 0.05) between both media (Figure 2). No morphological alterations were observed during microscopic analysis for both cultures (data not shown).

### 2.2. Effect of Adding a CD Lipid Mixture on In Vitro Proliferation of B. bovis-SF

**Figure 3:**
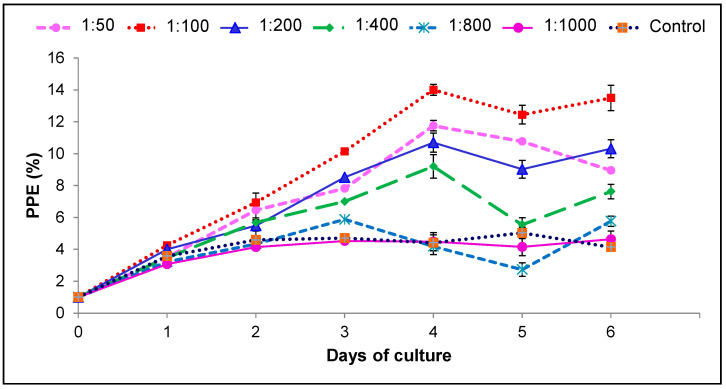
Addition of CD lipid mixture in VP-SFM enhances in vitro growth of *B. bovis*-SF. Values represent the mean from three separate experiments using triplicate wells for each test condition. PPE: percentage of parasitized erythrocytes. Subculturing was performed when PPE was above 4% and adjusted back to 1%.

The addition of a chemically defined lipid mixture diluted from 1:50 to 1:1000 in VP-SFM (*v*/*v*) was suitable for culturing *B. bovis* in vitro (Figure 3). However, the optimum dilution that maintained the in vitro growth of *B. bovis* was a 1:100 dilution of a CD lipid mixture with a maximum PPE of 13.9% on day 4. There were statistical differences (*p* < 0.05) compared to the other CD mixture dilutions.

### 2.3. B. bovis-SF Proliferation by Using VP-SFM with CD Lipid Mixture Addition

**Figure 4:**
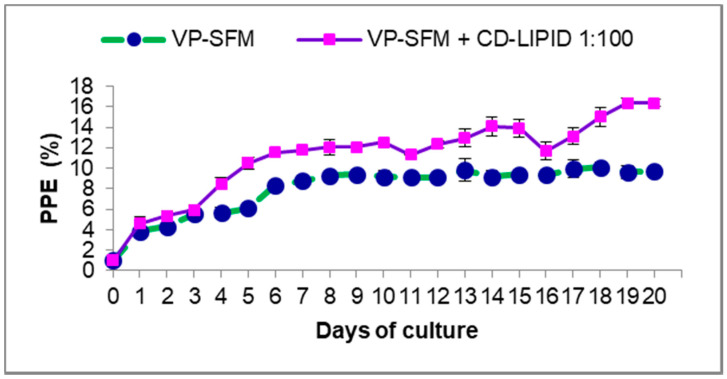
Evaluation of the VP-SFM culture medium with an optimal dilution (1:100) of CD lipid mixture on in vitro proliferation of *B. bovis*-SF. Values represent the mean from three separate experiments using triplicate wells for each test condition. PPE: percentage of parasitized erythrocytes. Subculturing was performed when PPE was above 4% and adjusted back to 1%.

The inclusion of a CD lipid mix (1:100) in the VP-SFM medium effectively enhanced the continuous proliferation of *B. bovis*-SF in vitro. During the first three days, comparing the VP-SFM medium with or without lipids showed that both treatments were similar (Figure 4). VP-SFM with a CD lipid mixture enhanced the PPE above 8%, with a maximum level of 16.39% vs. a maximum of 10% without lipids. There were statistical differences between the two treatments (*p* < 0.05).

### 2.4. Effect of VP-SFM Culture Medium Enriched with CD Lipid Mixture on the Proliferation in a Hollow Fiber Perfusion Bioreactor System

**Figure 5:**
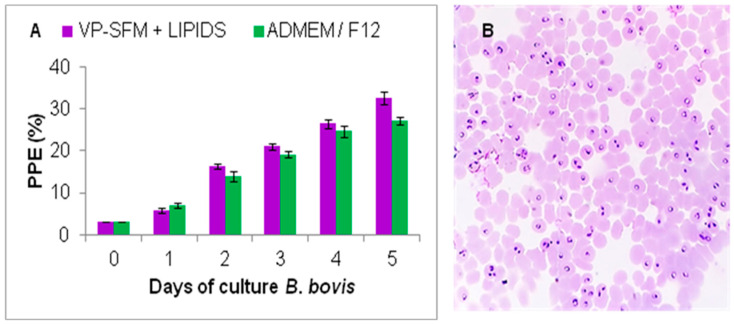
Large-scale in vitro proliferation of *B. bovis* in a hollow fiber perfusion bioreactor system (HFPBS). (**A**) Growth of *B. bovis* in VP-SFM supplemented with a CD lipid mixture in an HFPBS. Daily subcultures were made beginning at day 2 when the PPE exceeded 7% and adjusted back to 7% using a suspension of uninfected erythrocytes of 10% (*v*/*v*) in the culture medium. Values represent the mean from three separate experiments using triplicate wells for each test condition. PPE: percentage of parasitized erythrocytes. (**B**) Microscopic examination of Giemsa-stained smear of *B. bovis*-VP.

Based on the experiments described above, VP-SFM supplemented with the CD lipid mixture (1:100) was incorporated into a hollow fiber perfusion bioreactor system (HFPBS). The growth of *B. bovis* was compared to using ADMEM/F12 as a medium control. Although both media effectively scaled up the *B. bovis*-SF culture, the PPE values were higher with VP-SFM with the CD lipid mixture than with ADMEM-F12 (Figure 5). There were no significant differences between the treatments (*p* > 0.05). The highest PPE values were 32.59% and 27.19% for VP-SFM with the CD lipid mixture and ADMEM/F12, respectively. The parasites collected from both media showed a normal morphology on microscopic analysis (data not shown). After 30 days, infected erythrocytes were cryopreserved in liquid nitrogen, and parasites were unfrozen and successfully recovered for continuous *B. bovis* in vitro culture.

## 3. Discussion

This study demonstrated, for the first time, that a *B. bovis*-SF strain, previously adapted to the serum-free A-DMEMF/F12 medium, was successfully propagated in vitro using the VP-SFM culture medium free of components of animal origin. According to the manufacturer, the composition of basal medium VP-SFM contains no proteins, peptides, or other components of animal or human origin. VP-SFM was specifically designed for virus production [14,30,35]. Continuous in vitro culture of *B. bovis* using the VP-SFM or ADMEM/F12 culture medium was equally effective; both showed similar PPEs without any adaptation period required. Additionally, no morphological alterations were observed. The addition of a CD lipid mixture into the VP-SFM culture medium increased the PPE of *B. bovis*-SF. Similar results were reported by our group using VP-SFM as an alternative medium to grow *B. bigemina* [32]. The VP-SFM culture medium with a CD lipid mixture supported the growth of *B. bovis* for over 10 months (data not shown), indicating *B. bovis* can replicate in this medium for long cultivation periods. The VP-SFM medium contains essential components for the cultivation of *Babesia* spp. in vitro. One of these is putrescine; previous studies have confirmed that it is a key component that allows the rapid growth of *Babesia* spp. and other Apicomplexa parasites [1,11,36]. This is further supported by in silico analysis of the *B. bovis* genome that has shown the parasite cannot biosynthesize polyamines [37]. Other studies have described polyamines to be important for other protozoan parasites that cannot synthesize polyamines via *de novo* pathways. Thus, *T. gondii*, *Plasmodium falciparum*, *Trypanosoma brucei*, and *Leishmania donovani* acquire polyamines from the external environment [38,39,40,41]. Da Costa-Silva et al. (2012) described VP-SFM to be useful in producing tachyzoites of *T. gondii*, maintaining their infectivity and immunogenicity [31].

In this study, we demonstrated a rapid adaptation of a *B. bovis*-SF strain in a combination of VP-SFM and a CD lipid mixture with a growth rate of 16-fold in the culture plates. The parasitemia doubled four times in a 24-h period (1% to 2%, 2% to 4%, 4% to 8%, and 8% to 16%). Therefore, the parasites divided approximately every 6 h. This adaptation may represent selection of faster-growing genotypes and, consequently, result in the reduction in genetic variation within the culture-adapted parasites. We also showed a large proliferation and a higher volume of erythrocytes for growing *B. bovis* in vitro by combining VP-SFM and a CD lipid mixture in a perfusion bioreactor, reaching a PPE of over 30%. The addition of lipids improved the growth of the *B. bovis*-SF strain. It is well known that lipids are found in all living organisms in large quantities, as part of cell membranes and as an energy source. For apicomplexan parasites, lipids are essential for maintenance and survival. They are associated with the location and function of many proteins and are essential pathogenesis factors in different infectious diseases [42,43,44].

Previous studies suggested that the intraerythrocytic proliferation of apicomplexans such as *P. falciparum* requires many fatty acids that are obtained from the host plasma. Erythrocytes have negligible lipid synthesis and metabolism [44,45,46]. A previous report showed that in *T. gondii*, *de novo* parasitic acyl-lipid synthesis and recycling of host cell compounds coexist [47]. *Plasmodium* parasites obtain fatty acids either from the vertebrate host, from a mosquito vector, or by producing fatty acids by a *de novo*, type two fatty acid biosynthesis pathway (FAS-II) [48]. A recent report demonstrated that the FAS-II pathway is not required during the lytic cycle of *Toxoplasma* because of its elasticity in acquiring fatty acids [49].

We successfully cultivated *B. bovis* in vitro in a perfusion bioreactor using compounds free of animal origin and increased the erythrocyte volume. Previously, we reported the high proliferation of *B. bigemina* using the same culture conditions [32,36]. These are the only two reports on *Babesia* spp. cultured in a bioreactor [17]. Different bioreactors have been used successfully for the expansion of cell lines for cell therapy [50]. It has been suggested that the bioreactor facilitates the hydrodynamics and enables gas exchange and nutrient and waste removal. The bioreactor facilitates the cultivation of intraerythrocytic parasites and is an automated production method, providing a higher-quality biological material at reduced cost to produce live parasites [36]. Typically, production of live vaccines to control bovine babesiosis requires large numbers of splenectomized animals. In this study, only a single animal was used as the erythrocyte donor to generate large numbers of parasites using a bioreactor. Thus, in vitro cultivation of *Babesia* spp. in bioreactors represents an opportunity to reduce and replace the use of animals while producing sufficient biological material for diagnostics and live vaccines.

## 4. Materials and Methods

### 4.1. Microaerophilic Stationary-Phase Culture of B. bovis-SF

A Holstein Friesian five-year-old was used as an erythrocyte donor for *B. bovis* in vitro culture. The animal was purchased from a tick-free area in central Mexico and tested negative for brucellosis, tuberculosis, leucosis, IBR, BDV, *Anaplasma marginale*, and *Babesia* spp. The animal was handled according to approved animal care and use guidelines (NOM-062-ZOO-1999.2.2). Blood was obtained from the jugular vein, defibrinated with glass beads, and centrifugated at 450× *g* at 4 °C for 30 min. The serum and buffy coat were discarded. Erythrocytes were washed three times in A-DMEM/F12 containing antioxidants diluted at 1:1000 (Sigma-Aldrich, St. Louis, MO, USA). The final packed cell volume of the erythrocytes was 50% in A-DMEM/F12 + antioxidants and stored at 4 °C until use [17].

A *B. bovis*-SF strain previously adapted to proliferate in serum-free medium was used in this study [10,11]. The MASP methodology previously defined was carried out as described [23]. The in vitro culture started with a percentage of parasitized erythrocytes (PPE) of 1%, in a 10% suspension of erythrocytes in A-DMEM/F12 medium (*v*/*v*), under a humidity-saturated atmosphere with a mixture of 90% N_2_, 5% O_2_, and 5% CO_2_ at constant pressure, in a final volume of 1000 µL. The medium was replaced daily when the culture of *B. bovis*-SF was in logarithmic growth [32]. Subculturing was performed when any culture exceeded a PPE of 4% and adjusted to 1% by the addition of uninfected erythrocytes. If the PPE was ≤ 1%, attempts to recover the culture were performed by diluting the culture 1:2 with fresh erythrocytes.

### 4.2. Culture Media and Supplements

Commercially available culture media were evaluated for *B. bovis* in vitro growth, including VP-SFM AGT™ (VP-SFM) (GIBCO^®^, Grand Island, NE, USA), CD-CHO (GIBCO^®^), CD-Hydrolyzed (Sigma-Aldrich), CD-CHO (Sigma-Aldrich), Hybrigro SF Medium (SFM) (CORNING^®^ Inc., Kansas city, KS, USA), and Advanced-DMEM/F12 (ADMEM/F12) (GIBCO^®^). Each culture medium was buffered with 25 mM 2-[(2-hydroxy-1, 1-bis(hydroxymethyl) ethyl) amino] ethane sulfonic, N-[Tris (hydroxymethyl) methyl]-2-aminoethanosulphonic (TES) (Sigma-Aldrich), and 25 mM L-glutamine (GIBCO^®^) was added. A mixture of antioxidants was added to all culture media as described above and supplemented with a commercially available chemically defined lipid concentrate (CD-lipid mixture, GIBCO^®^). The CD lipid mixture contained 2 μg/mL arachidonic and 10 μg/mL each of linoleic, linolenic, myristic, oleic, palmitic, and stearic acids diluted in the media at 1:50, 1:100, 1:200, 1:400, 1:800, and 1:1000. The media were adjusted to pH 6.8 and filter sterilized through a 0.22-µm pore size membrane (Millipore St. Louis, MO, USA).

### 4.3. Selecting an Optimal Culture Medium Using Medium Free of Components of Animal Origin for the In Vitro Proliferation of B. bovis-SF

VP-SFM, CD-CHO, CD-Hydrolyzed, CD-CHO, SFM, and ADMEM/F12 were used to culture *B. bovis*-SF. Thin blood smears were made from each well, fixed with absolute methanol, and Giemsa stained to monitor parasite proliferation. The PPE was determined by counting 5000 erythrocytes for each smear. Three separate experiments were carried out utilizing triplicate wells for each test condition [11].

### 4.4. Effect of Different Concentrations of a CD Lipid Mixture on In Vitro Proliferation of B. bovis-SF

After determining that VP-SFM supports the growth of *B. bovis* in culture, we added a commercially available CD lipid mixture containing a completely defined combination of saturated and unsaturated fatty acids to determine if the growth of *B. bovis* was enhanced. *B. bovis*-SF continuously proliferated in VP-SFM culture medium, and the effect of lipids was evaluated using six dilutions of 1:50, 1:100, 1:200, 1:400, 1:800, and 1:1000 (*v*/*v*) of the CD lipid mixture.

### 4.5. Effect of VP-SFM Culture Medium Enriched with CD Lipid Mixture on the Proliferation of B. bovis-SF in a Hollow Fiber Perfusion Bioreactor System (HFPBS)

A hollow fiber perfusion bioreactor system (HFPBS) (FiberCell^®^System, New Market, MD, USA) was used during the cultivation of *B. bovis* to increase the volume of erythrocytes and PPE. Inside the HFBPS cartridge, 5 mL of erythrocytes with 3% PPE was placed, and 35 mL of uninfected erythrocytes was added. Subsequently, 350 mL of VP-SFM supplemented with a 1:100 dilution of CD lipid mixture circulated in a continuous flow through the bioreactor using a peristaltic pump. The culture was monitored, and the medium was replaced daily according to a previously described methodology [11]. *B. bovis* culture was adjusted daily to a 7% PPE by the addition of uninfected erythrocytes.

### 4.6. Statistical Analysis

Data were analyzed as percent differences in parasitemia for *B. bovis*-SF in vitro cultures using one-way ANOVA, followed by Dunnett’s test to compare means of different experimental groups with the mean of a control group. Additionally, an independent *t*-test was performed to compare VP-SFM vs. ADMEM-F/12 in plates and VP-SFM + LIPIDS vs. ADMEM/F12 in a bioreactor.

## 5. Conclusions

The VP-SFM medium supplemented with a mixture of lipids allowed the continuous in vitro proliferation of *B. bovis* at small and large scales. In bovine babesiosis endemic regions, it is advantageous to produce attenuated vaccines using a lipid-supplemented medium free of animal products with expansion in a bioreactor for large scales. Furthermore, the elimination of components of animal origin from the culture medium and large-scale production of parasites could potentially reduce and replace the use of animals to produce reagents for diagnostic and live vaccine purposes to control bovine babesiosis in endemic areas.

## Data Availability

Not Applicable.
